# The BC Radon Data Repository (BCRDR) and BC Radon Map: Integrating disparate data sources for improved public health communication

**DOI:** 10.17269/s41997-024-00895-5

**Published:** 2024-05-28

**Authors:** Jeffrey Trieu, Cheryl Young, Phuong D. M. Nguyen, Anne-Marie Nicol, Sarah B. Henderson, David McVea

**Affiliations:** 1 Environmental Health Services, British Columbia Centre for Disease Control, Vancouver, BC Canada; 2 Faculty of Health Sciences, Simon Fraser University, Burnaby, BC Canada; 3 National Collaborating Centre for Environmental Health, Vancouver, BC Canada; 4 School of Population and Public Health, University of British Columbia, Vancouver, BC Canada

**Keywords:** Radon, Data aggregation, Data anonymization, Data visualization, Geographic mapping, Radon, agrégation de données, anonymisation de données, visualisation de données, cartographie géographique

## Abstract

**Setting:**

The potential for exposure to indoor radon varies dramatically across British Columbia (BC) due to varied geology. Individuals may struggle to understand their exposure risk and agencies may struggle to understand the value of population-level programs and policies to mitigate risk.

**Intervention:**

The BC Centre for Disease Control (BCCDC) established the BC Radon Data Repository (BCRDR) to facilitate radon research, public awareness, and action in the province. The BCRDR aggregates indoor radon measurements collected by government agencies, industry professionals and organizations, and research and advocacy groups. Participation was formalized with a data sharing agreement, which outlines how the BCCDC anonymizes and manages the shared data integrated into the BCRDR.

**Outcomes:**

The BCRDR currently holds 38,733 measurements from 18 data contributors. The repository continues to grow with new measurements from existing contributors and the addition of new contributors. A prominent use of the BCRDR was to create the online, interactive BC Radon Map, which includes regional concentration summaries, risk interpretation messaging, and health promotion information. Anonymized BCRDR data are also available for external release upon request.

**Implications:**

The BCCDC leverages existing radon measurement programs to create a large and integrated database with wide geographic coverage. The development and application of the BCRDR informs public health research and action beyond the BCCDC, and the repository can serve as a model for other regional or national initiatives.

**Supplementary Information:**

The online version contains supplementary material available at 10.17269/s41997-024-00895-5.

## Résumé

**Lieu:**

Le potentiel d’exposition au radon à l’intérieur des bâtiments varie beaucoup d’une région à l’autre de la Colombie-Britannique en raison de la géologie variée. Les particuliers peuvent avoir du mal à comprendre leur risque d’exposition, et les organismes, à comprendre l’utilité des programmes et des politiques populationnels pour atténuer le risque.

**Intervention:**

Le BC Centre for Disease Control (« le Centre ») a créé un organe d’archivage, le BC Radon Data Repository (BCRDR), pour faciliter la recherche, l’information, la sensibilisation du public et l’action liées au radon dans la province. Le BCRDR totalise les relevés du radon à l’intérieur des bâtiments pris par les organismes gouvernementaux, les professionnels et les organismes de l’industrie, ainsi que les groupes de recherche et de revendication. La participation est officialisée par un accord de partage de données qui décrit comment le Centre anonymise et gère les données communes du BCRDR.

**Résultats:**

Le BCRDR contient actuellement 38 733 relevés de 18 contributeurs de données. Il continue de croître, avec de nouveaux relevés venant de contributeurs existants et l’ajout de nouveaux contributeurs. Il a servi, entre autres, à créer une carte du radon interactive en ligne pour la Colombie-Britannique, avec des résumés des concentrations régionales, des messages d’interprétation du risque et des informations de promotion de la santé. Sur demande, les données anonymisées du BCRDR sont également disponibles pour diffusion externe.

**Conséquences:**

Le Centre a exploité les programmes de prise de relevés du radon existants pour créer une grande base de données intégrée ayant une vaste couverture géographique. Le développement et les applications du BCRDR éclairent la recherche et l’action en santé publique au-delà du Centre, et l’organe d’archivage peut servir de modèle pour d’autres initiatives régionales ou nationales.

## Setting

Radon (^222^Rn) is a colourless, odourless, and tasteless carcinogenic gas formed during the natural radioactive decay of uranium in rock and soil. Radon gas quickly dilutes in outdoor air but can accumulate within confined indoor spaces. Long-term inhalation exposure can lead to the development of lung cancer via alpha-radiation emitted from radon and its progeny. Pooled epidemiologic studies suggest that long-term exposure to indoor radon is the second leading cause of lung cancer globally and the leading cause among non-smokers (World Health Organization, 2009).

Measuring indoor radon is easy and relatively inexpensive with a variety of widely available testing devices. Health Canada recommends taking action to mitigate indoor levels of radon if an average concentration of 200 Bq/m^3^ or higher is found from a minimum 3-month test (Health Canada, 2017). This falls within the World Health Organization’s recommendation to set national action levels between 100 and 300 Bq/m^3^ (World Health Organization, 2009). Given the linear dose–response relationship with lung cancer risk, the Canadian 200 Bq/m^3^ action level does not represent a binary risk threshold (World Health Organization, 2009). Therefore, individuals should strive to reduce indoor levels of radon to as low as reasonably achievable (ALARA). There are multiple effective mitigation strategies that range in cost and complexity. Mitigation can be as simple as sealing ground contact entry points or as involved as installing a depressurization and ventilation system (Health Canada, 2013).

Unique differences in building design, deterioration over time, and ventilation mean that indoor radon levels can vary from building to building, even in the same neighbourhood. Still, there are macro-level patterns of indoor radon variation in British Columbia (BC). BC has five tectonic belts with the eastern belts having more favourable conditions for uranium deposits and, therefore, more potential for surface level radiation (Radon Environmental, 2019; WorkSafe BC, 2009). Results from a national survey of indoor radon measurements supported the observations from these studies of geologic radon potential, finding elevated rates of indoor radon concentrations in the northern and interior regions of BC (Health Canada, 2012). This has led to new building codes that differ across the province (Government of British Columbia, 2023).

In BC, radon measurement data are held by different entities that have conducted testing campaigns or studies, sold measurement devices, or provided measurement services. Each individual dataset holds valuable information, but none is sufficient in isolation to adequately assess radon exposure risk across the province at a localized scale. To address this issue, the BC Centre for Disease Control (BCCDC) established the BC Radon Data Repository (BCRDR)—an aggregation of BC indoor radon measurements from volunteer contributing organizations. Organizations share their radon measurement data with the BCCDC, which anonymizes and integrates them using standardized methods outlined in a data sharing agreement (DSA). The BCCDC uses the BCRDR data holdings to create the online, interactive BC Radon Map, which includes regional concentration summaries, risk interpretation messaging, and health promotion information (https://bccdc.shinyapps.io/bcradonmap/). This article describes the technical and operational development of the BCRDR and BC Radon Map.

## Intervention

Radon data are held by a range of entities, not all of which are familiar with sharing data for public health use. Our first step in establishing the BCRDR was to develop a DSA to establish our intent to act as good stewards of an integrated database that facilitates public health research and action, while preventing identification of any individual person or building unit. The DSA formalizes this with the following core principles:

- Data providers remain owners of their data. A data provider can end participation at any time, and their data will be removed from the BCRDR and destroyed.
- BCCDC stores and manages all BCRDR data using the same security protocols as other sensitive data holdings.
- BCCDC anonymizes all raw data received from data providers before storing it in the BCRDR, eliminating person- and building-level unique identifiers.
- Any party requesting BCRDR data will submit a formal request to the BCCDC, which will review it and only approve requests that address health protection research and action. BCCDC will release only the minimum data needed to meet the stated goals.
- All publications or distributed materials using BCRDR data will only show de-identified or summary level information and must be reviewed by BCRDR data stewards for compliance.

We asked data providers to share the type of testing device used; the device serial number; the start and end date of the test; the average result concentration; the geographic location of the measurement site; and any contextual building information. Due to differences in the geographic information available from various providers and their willingness to share, we adopted a flexible approach and accepted complete addresses, 6-digit postal codes, 3-digit forward sortation areas (FSA), or city names. When available and not limited by existing data sharing policies, we encouraged sharing complete addresses, highlighting our data anonymization and handling practices to alleviate any potential concerns. We did not solicit any information related to building occupants. We retained only unique measurements that included, at minimum, a result concentration and a location that could be anonymously mapped.

**Fig. 1:**
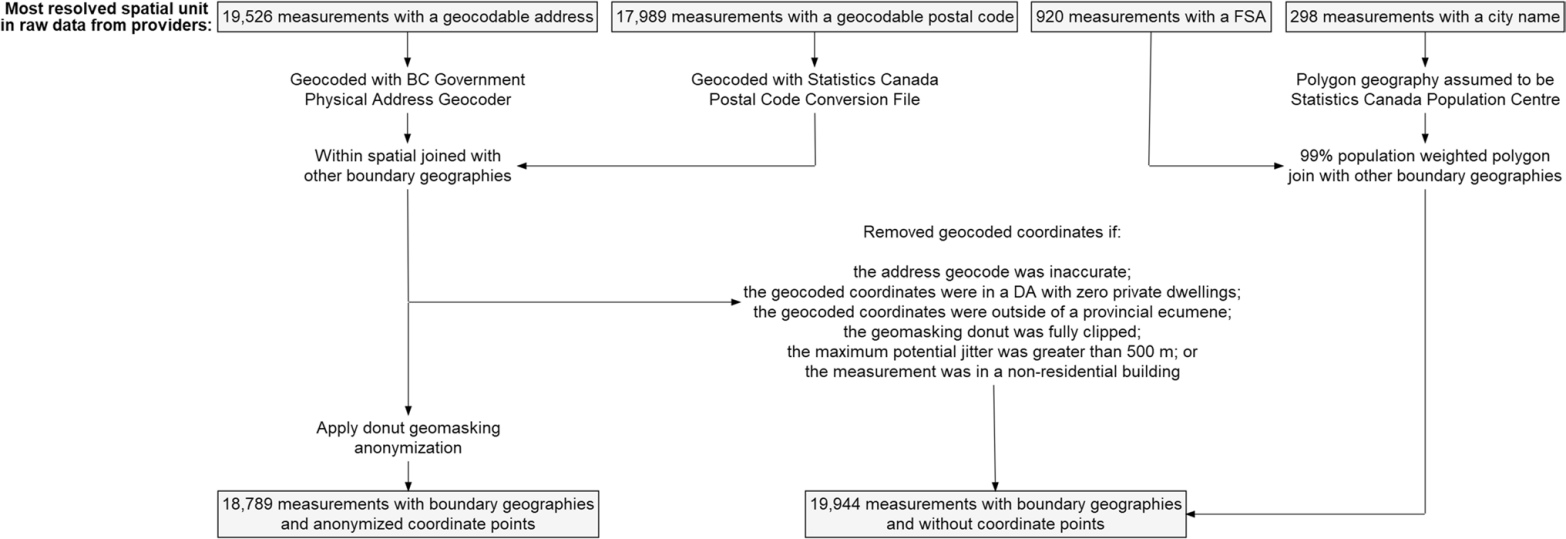
Spatial data management flowchart of the BC Radon Data Repository. Additional data management details can be found in the supplementary material (SM1)

We processed the provided address, postal code, FSA, or city name for each measurement site into anonymized point coordinates and/or boundary geographies (Fig. 1). These boundary geographies (and their number of polygons) were health authorities (HA; *n* = 5), health service delivery areas (HSDA; *n* = 16), local health areas (LHA; *n* = 89), community health service areas (CHSA; *n* = 218), FSA (*n* = 191), and legally defined administrative boundaries for municipalities (*n* = 160) and regional districts (*n* = 28).

We geocoded addresses and postal codes to point coordinates using BC’s Physical Address Batch Geocoder and Statistics Canada’s 2018 Postal Code Conversion File, respectively, and assigned them to the boundary geographies within which their geocoded points were located. We then applied a donut geomasking technique to anonymize the geocoded points, where coordinates would be displaced from their original location at a distance inversely correlated to the underlying residential dwelling density (Hampton et al., 2010).

To retain spatial validity, we limited the displacement to within the original coordinate’s CHSA and did not anonymize coordinates that could be displaced further than 500 m from their original location. This donut geomasking protocol was not practical if the geocoded coordinate was located in a dissemination area with a dwelling count of zero, the geocoded coordinate was located outside a 1 km buffer around the provincial ecumene (i.e., inhabited land area) (Smith, 2011), or the geomasking donut was fully outside of the geocoded coordinate’s CHSA. If any of these criteria were met, we did not retain the coordinate data and only kept the boundary geography spatial units for inclusion in the BCRDR. We also assumed non-residential buildings to be at high risk of re-identification, despite geomasking, and integrated these into the BCRDR with only the boundary geography spatial units. Additional data management details can be found in the supplementary material (see SM1).

Using BCRDR data, we developed the BC Radon Map (https://bccdc.shinyapps.io/bcradonmap/)—an R Shiny choropleth map displaying summary statistics by the four provincial health geographies and two administrative local government boundaries. In all regions with at least 20 unique residential building measurements, we display the 50th and 95th percentile of residential radon concentrations, as well as percentage of homes in different radon concentration categories that align with established national and international guidelines for radon mitigation (Health Canada, 2017; World Health Organization, 2009). We also reinforce the message that regional estimates do not replace the value of testing individual buildings, and we provide information on where to purchase a testing device and how to mitigate exposure in homes. We display simplified messages about estimated lifetime lung cancer risks associated with a range of lifetime radon exposures, for both smokers and non-smokers (Chen, 2005). Our communication goal was two-fold: to show how lung cancer risk increases with increasing radon exposure and how smoking impacts lung cancer risk in a multiplicative synergistic fashion.

## Outcomes

We reached out to all organizations and professional entities that we knew held radon measurement data in BC. Some potential data providers declined participation, citing privacy policies or consent forms that did not allow for data sharing. In most cases, we were able to leverage existing relationships through the BCCDC’s past radon health protection work. At the time of publication, the BCRDR had 19 data contributors spanning government agencies, industry professionals and organizations, and research and advocacy organizations (Table 1). All finalized agreements cover data collected into the future. Therefore, the BCRDR holdings will grow over time.

**Table 1:** Aggregate counts of data providers to the BC Radon Data Repository, as well as measurement counts and percentages of total 38,259 repository measurements

	Provider count	Measurement count (%)
Industry professionals and organizations	8	15,574 (40%)
Government agencies	7	14,256 (37%)
Research and advocacy organizations	4	8906 (23%)

At the time of publication, the BCRDR held 38,733 measurements from 17,713 known unique buildings (46%) (Table 2). There were an additional 2686 measurements for which we could not confirm the uniqueness of the building. If all these measurements were from unique buildings, the measurements at time of publication would be from 20,399 unique buildings (53%). A total of 17,335 measurements were from non-residential settings such as schools and health care sites (45%) and 21,398 measurements were from residential settings (55%). We successfully geocoded 20,931 residential measurements (98%) from their provided addresses or postal codes and successfully anonymized 18,789 (88%) (Fig. 1). 98% of measurements have a health geography, regional district, or FSA boundary geography. Municipality polygons do not cover the extent of the province and therefore have lower data availability at 84%. Only 121 measurements were done with a continuous testing device, whereas 35,868 were done with an alpha track detector (93%). The majority of measurements were taken either on the main ground floor (*n* = 16,113, 42%) or in the basement (*n* = 8495, 22%). Contextual information about the buildings was limited.

**Table 2:** BC Radon Data Repository descriptive case counts and percentages

	Count (%)
Total observations	38,733 (100%)
Known unique buildings	17,713 (46%)
Presumed unique buildings	20,399 (53%)
Spatial data availability	
With jittered point geographies	18,789 (49%)
With a municipality	32,522 (84%)
With a regional district	37,871 (98%)
With a forward sortation area	38,659 (99.8%)
With a community health service area	37,906 (98%)
With a local health area	38,091 (98%)
With a health service delivery area	38,398 (99%)
With a health authority	38,506 (99%)
Testing date availability	
Start and end date	35,411 (91%)
Start year	37,575 (97%)
Building type	
Residential	21,398 (55%)
Non-residential	17,335 (45%)
Test floor	
Upper floor	1126 (3%)
Main floor	16,113 (42%)
Basement	8495 (22%)
Crawl space	28 (0%)
Lowest floor	83 (0%)
Unknown	12,888 (33%)
Testing device	
Alpha track detector	35,868 (93%)
Continuous monitoring device	121 (0%)
Unknown	2744 (7%)
Test occurrence	
Pre-mitigation	12,148 (31%)
Post-mitigation	550 (1%)
Unknown	26,035 (67%)
Presence of an AC system	
Yes	543 (1%)
No	2939 (8%)
Unknown	35,251 (91%)
Heating system	
Forced air	3710 (10%)
Radiant water	286 (1%)
Baseboard	150 (0%)
Multiple systems	1230 (3%)
Other	692 (2%)
Unknown	32,665 (84%)
Structural building type	
Detached	5765 (15%)
Semi-detached or attached	507 (1%)
Condo/apartment	633 (2%)
Other	285 (1%)
Unknown	31,543 (81%)
Build year	
1920 or before	169 (0.4%)
1921 to 1945	371 (1%)
1946 to 1960	960 (2%)
1961 to 1970	1155 (3%)
1971 to 1980	2316 (6%)
1981 to 1990	1270 (3%)
1991 to 2000	1581 (4%)
2001 to 2010	1389 (4%)
2011 or after	932 (2%)
Unknown	28,590 (74%)

**Fig. 2:**
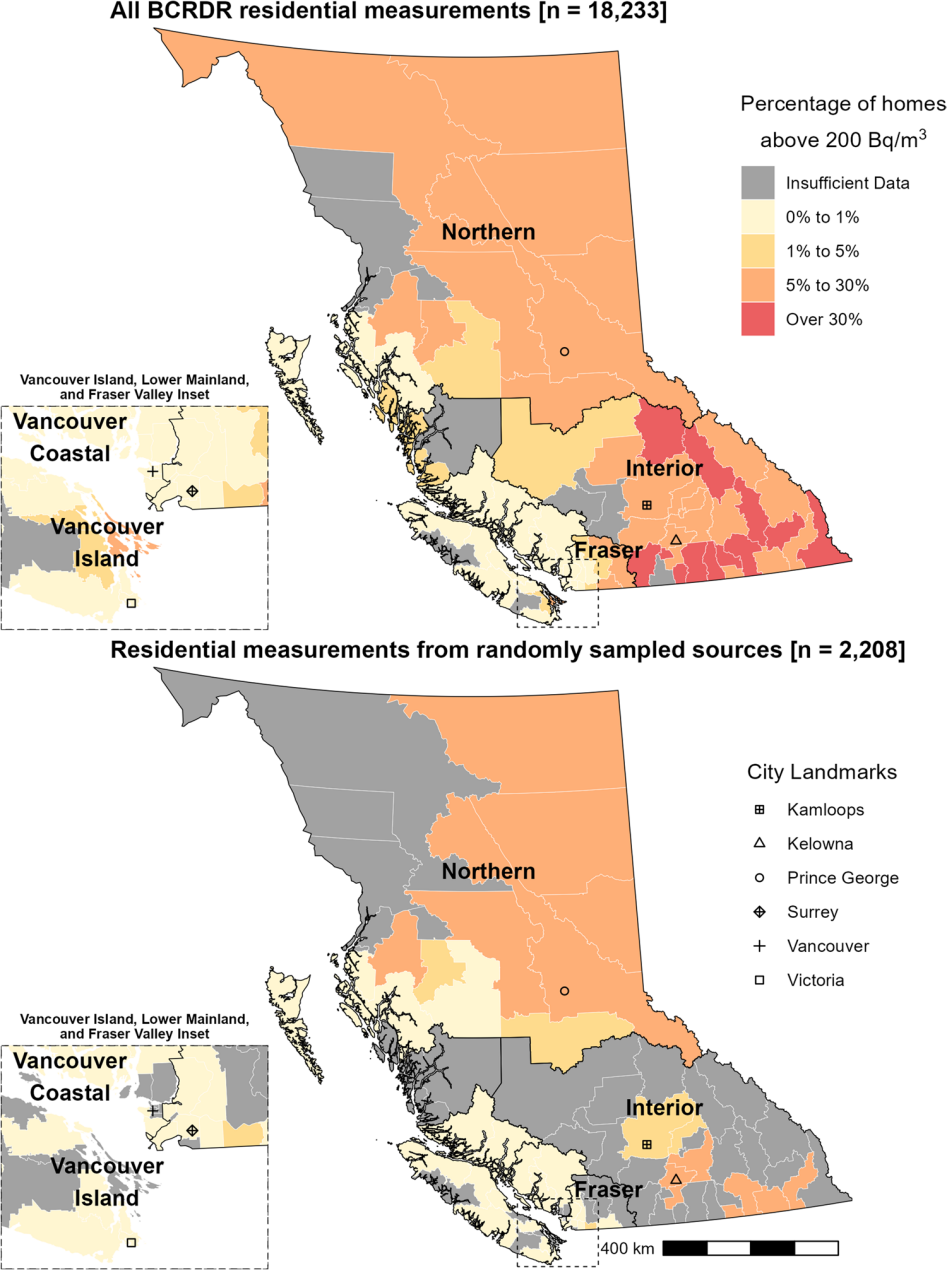
Local Health Area (LHA) map of percentage of homes above 200 Bq/m^3^. These are plotted for all residential measurements in the BC Radon Data Repository (BCRDR, top) and only residential measurements randomly sampled in Health Canada’s Cross-Canada Survey and Radon and Thoron Data from Canadian Homes survey (bottom). Results for LHAs with less than 20 unique residential building measurements were not plotted. Figure updates with additions to the BCRDR after the time of publication can be found at https://bccdc.shinyapps.io/bcradonmap/

At the time of publication, 79 of 89 LHAs had at least 20 unique residential measurements to generate summary statistics (Fig. 2). Among Interior Health Authority’s 31 LHAs, 27 had over 5% of homes with more than 200 Bq/m^3^. Among Northern Health Authority’s 17 LHAs, 9 had over 5% of homes with more than 200 Bq/m^3^. Among Fraser, Vancouver Coastal, and Vancouver Island Health Authorities’ 41 LHAs, 3 had over 5% of homes with more than 200 Bq/m^3^. More and up-to-date regional summaries can be found at https://bccdc.shinyapps.io/bcradonmap/.

## Implications

The BCRDR integrates province-wide radon measurement data from several voluntary contributors into a central repository to be used for public health research and action. The core principles of the BCRDR are outlined in its DSA, which defines roles and responsibilities, organizational security practices, anonymization techniques, and limitations on data use. The BCCDC did not have a standard data sharing process for radon measurement data prior to the development of the BCRDR, though we did receive data on an ad hoc basis, amounting to 3867 measurements (Rauch & Henderson, 2013). At the time of publication, the BCRDR holds nearly ten times as many measurements. We estimate that the data holdings will increase by 2000 measurements annually from the ongoing data collection from existing data providers, with the ability to increase even more with new providers.

A well-designed random sampling strategy is the best way to estimate unbiased population exposure to radon. However, it is highly resource-intensive to execute this at scale with representative sampling at smaller geographies. The BCRDR leverages existing radon measurement programs, thereby reducing resource costs, to create a large integrated data holding that can support more localized action and understanding of radon exposure in the province. At the time of publication, the BCRDR leverages 18,233 measurements to report summary statistics in 79 of 89 LHAs (Fig. 2). Among the current BCRDR data sources, only the Health Canada surveys used a random sampling approach (Chen et al., 2015; Health Canada, 2012). If we were to limit to only these 2208 randomly sampled measurements, summary statistics could only be reported in 42 LHAs. This approach would exclude the LHAs where indoor radon exposure risk is highest, in the southern and eastern interior. It would also exclude local hot spots in eastern Fraser Valley and Cowichan Valley in the otherwise broader lower risk regions of Vancouver Island and the coastal mainland.

**Fig. 3:**
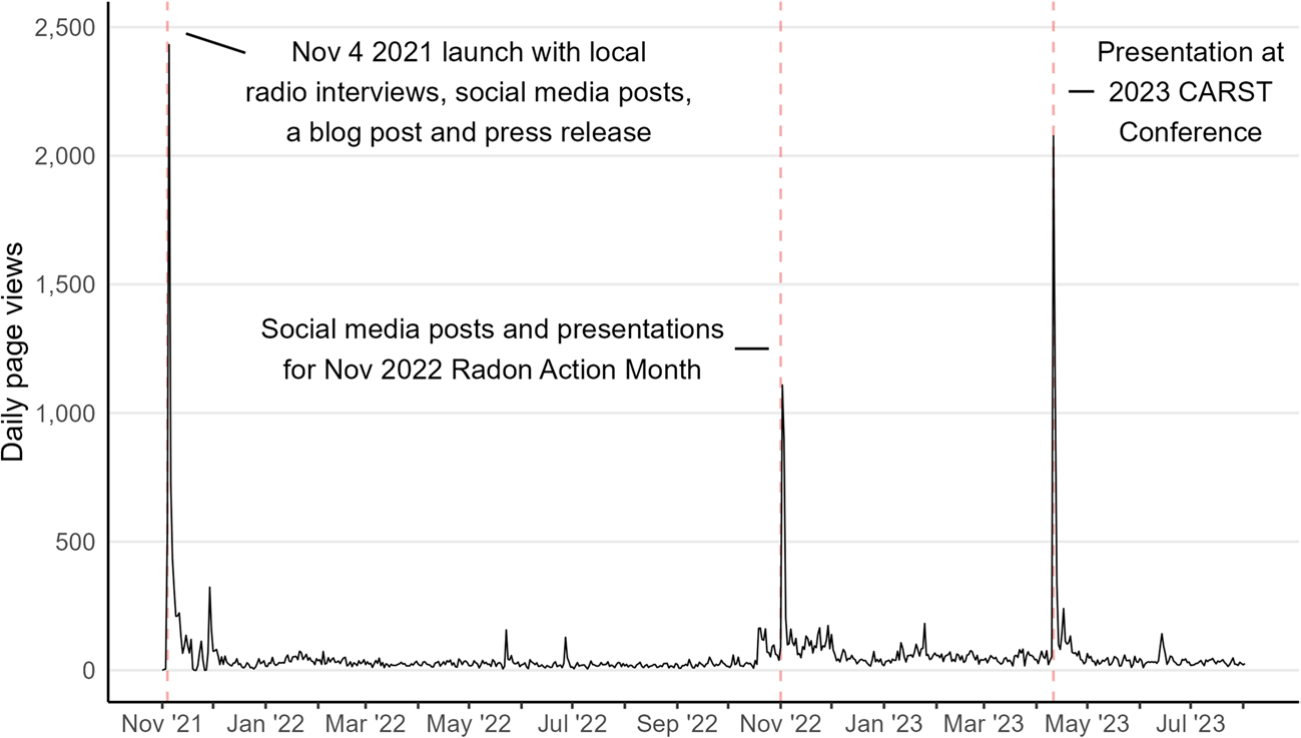
Daily page views to the BC Radon Map from November 2021 through July 2023 (https://bccdc.shinyapps.io/bcradonmap/)

Public radon risk maps increase perceived risk and increase willingness to test for radon (Timmons & Lunn, 2023). Further, they can be used by stakeholders in the development of policies and programs, showing where radon interventions may be prioritized. When we launched the BC Radon Map in November 2021, it was accompanied by promotional efforts, such as social media posts and a press release. This helped generate nearly 5000 page views in the first week (Fig. 3). The map received a similar number of page views in November 2022 when we put out social media posts for Radon Awareness Month and over 4000 during the week of the 2023 Canadian Association of Radon Scientists and Technologists conference when we presented at this venue. Other than these three periods, the map averages nearly 40 page views per day, suggesting that the product has value for stakeholders and the public beyond our promotional efforts. Future work can explore how the BC Radon Map and its design choices impact user attitudes and behaviours associated with radon understanding and commitment to action.

Data sharing with the BCRDR is completely voluntary. Therefore, an effective data anonymization framework is essential for building trust with data contributors, even if radon measurement data are not considered personal information (Quastel, 2021). We followed an organizational policy to define the donut geomasking calculation for magnitude of displacement. We did not retain anonymized coordinates for non-residential buildings due to high re-identification risk given that the geomasking method only considers private dwelling density to inform the magnitude of jitter. Future work can explore the use of cadastral files (i.e., land property geospatial data) for non-residential building geomasking. Beyond spatial data anonymization, BCRDR data are made non-identifiable through organizational data security practices such as storage on a secure network, managed internal access, and external release only for application-approved research and health protection projects.

The BCRDR is a data compilation effort, which means we cannot evaluate the quality of the source data or its collection. Another limitation of the BCRDR is the sparse information about individual building characteristics, which could be used to contextualize measurement results (Khan et al., 2021). Mitigation status was also not included in most compiled measurements. Therefore, mitigation prevalence cannot be evaluated with BCRDR as it presently exists and unknown post-mitigation measurements may bias summary statistics downwards. Coordinates derived from rural 6-digit postal codes (which have 0 as their second digit) have relatively low positional accuracy (Khan et al., 2018). However, only 1178 measurements (8%) with anonymized coordinates in the BCRDR were geocoded from rural postal codes. If this reduced spatial validity is a concern for a particular analysis, these measurements could be excluded entirely or excluded in a sensitivity analysis subset. The BCRDR mostly includes measurements using an alpha track detector and lacks measurements from continuous radon monitors. Alpha track detectors require users to send the detector to the manufacturer’s laboratory for analysis, which is a touch point to create data that are shareable by those who procure, organize, or offer measurement services. Future work can explore data sharing with manufacturers of continuous radon detectors.

The size and spatial resolution of the BCRDR allow for analysis and reporting on radon exposure risk at a scale previously unprecedented in Canada, providing more localized information for individuals and public health partners. Anonymized data are available for external release, so the BCRDR can inform the research and health protection work of others, without duplicating potentially cost-prohibitive data acquisition and cleaning work. Other jurisdictions and program areas could benefit from the BCRDR’s framework for exposure data integration, anonymization, and application.

## Implications for policy and practice

What are the innovations in this policy or program?

- The BC Radon Data Repository (BCRDR) has formalized data anonymization, management, and access protocols using a data sharing agreement to minimize privacy concerns and solicit contributions from government, private industry, and non-government organizations.
- The BCRDR applies a donut geomasking protocol to geocoded address and postal code coordinates, which allows for anonymized, yet spatially valid, use of location data.
- The size and spatial resolution of the BCRDR allow for analysis and reporting on radon exposure risk at a scale previously unprecedented in Canada, providing more localized information for individuals and public health partners.

What are the burning research questions for this innovation?

- Additional research is required to evaluate how the BC Radon Map is used and interpreted to ensure the product’s content and design achieve the goal of increasing radon awareness, improving understanding of radon exposure and lung cancer risk, and motivating individuals to act.
- The findings of said evaluation research could be communicated to prospective data providers to increase the likelihood of data sharing, thereby improving ecological risk estimations and potential uses of the data.
- Future work can explore whether the BCRDR’s framework can be applied to other data relevant to public health that are held by multisector parties.

## Supplementary Information

Below is the link to the electronic supplementary material.

Supplementary file1 (DOCX 23 KB)

## Data Availability

Anonymized data are available upon request.
