# Supplementary material for: The BC Radon Data Repository (BCRDR) and BC Radon Map: Integrating disparate data sources for improved public health communication
Source: Can J Public Health. 2024 May 28;115(4):680–7. doi: 10.17269/s41997-024-00895-5 (PMC11303615; doi:10.17269/s41997-024-00895-5)
Supplement: Supplementary file 1 — Supplementary file1 (DOCX 23 KB) [file 41997_2024_895_MOESM1_ESM.docx]

**Supplemental Material** – Additional data management details.

We performed all data management and analysis in the R statistical computing environment (R Core Team, 2021).

The BC Physical Address Geocoder matches address strings to either a building unit, building overall, block face, street, city or town, or province. The resulting coordinates can be derived from sources with varying degrees of positional accuracy, such as satellite imagery with ≤ 1 metre resolution, property boundary polygons, or interpolation from a block face address range. If the resulting coordinates did not match a building with the positional accuracy of at least a property boundary, we considered geocoding to have failed and instead geocoded the postal code, if present.

To donut geomask the geocoded coordinates, we calculated the private dwelling density from each coordinates’ underlying 2021 census dissemination block. If this was zero, we used the dissemination area. We followed an organizational policy to define the inner and outer donut radii as two and five times the square root of the inverse private dwelling density. We examined the anonymization effectiveness of these parameters via *k*-anonymity, where *k* is the estimated number of private dwellings in the geomasking area (Sweeney, 2002). To calculate a measurement’s *k*-anonymity score, we summed the private dwelling counts from all 2021 census dissemination blocks that had any polygon overlap with the geomasking donut. This provided an unweighted *k*-anonymity score. For a weighted *k*-anonymity score, we scaled the private dwelling count to the proportion of area overlap with the geomasking donut, then summed the scaled values. Among measurements with retained anonymized coordinates, the median weighted private dwelling *k*-anonymity score was 63 (99^th^ percentile range: 6 – 1361). Low weighted *k*-anonymity scores were likely due to area overlap scaling. 95% of measurements with a below median weighted *k*-anonymity score had an unweighted score of 100 or more and all but 3 had an unweighted *k*-anonymity score of 16 or more. These 3 outlier measurements had small jittering donuts and were geocoded to the coast, meaning their jittering donut was further clipped to the provincial coastal boundary. This suggests that the created geomasking donuts included a sufficient number of other private dwellings to reasonably anonymize the location of the original dwelling.

Some rural postal codes coordinates are central to a very large delivery area. Inherently, these points have too much spatial error to be reliably used as points. We took a more conservative approach and did not retain coordinates values for any measurements with geocoded coordinates that fell outside of a 1 km buffer of the provincial ecumene (i.e., inhabited land area). The provincial ecumene used considers census, geographic, and land use data (Smith, 2011). The 1 km buffer was chosen as it covers most within-city inhabitable areas such as city parks.

For measurements with solely city names, we assumed they fell within the polygon geography of Statistics Canada Population Centres. For these and FSAs, we mapped them to other boundary geographies they had at least 99% population weighted area overlap with.

Some source data had explicit indication of multiple measurements within one building (e.g., a measurement done pre- and post-mitigation in a home). When not available, we considered measurements with matching geocoded address string coordinates as measurements within the same building. We assigned same building measurements a numeric building ID value and gave them the same geomasked coordinates.

We removed all suspected duplicate measurements based on detector serial number, testing dates and location, and the result concentration value.

We report summary statistics with one measurement per building, on the lowest floor in a pre-mitigated state. If there was no explicit indication of mitigation status in the source data, we used the earliest higher measurement value. If more than one measurement still remained, we choose the lowest testing floor value via this stepwise procedure: 1) if all measurements were taken on unknown floors, select one at random; 2) if measurements were taken on an unknown floor and main or upper floors, select one at random; 3) if measurements were taken on an unknown floor and lowest floor, crawl space, or basement, use one value from the lowest floor, crawl space or basement measurement in this order; and 4) otherwise, use one value with the following hierarchy: lowest floor, crawl space, basement, main floor, and then upper floor. The "Main Floor" is the ground level or first floor of the building, not including a basement or crawl space. The "Upper Floor" is any floor above the Main Floor. The "Lowest Floor" is if the specific floor level is not indicated however there was explicit mention that the test occurred in the lowest floor of the building.

**REFERENCES**

R Core Team. (2021). *R: A language and environment for statistical computing. R Foundation for Statistical Computing*. <https://www.r-project.org/>

Smith, A. (2011). *Mapping the population ecumene of British Columbia: A multilevel binary dasymetric GIS methodology | IRMACS Thematic Year 2011*.

Sweeney, L. (2002). K-Anonymity: A Model for Protecting Privacy. *International Journal of Uncertainty, Fuzziness and Knowledge-Based Systems*, *10*(05), 557–570. <https://doi.org/10.1142/S0218488502001648>
